# Social contact impacts physical activity and sedentary behavior among older adults in Japan due to COVID-19

**DOI:** 10.1186/s12877-022-03188-z

**Published:** 2022-06-08

**Authors:** Naoto Otaki, Miyuki Yokoro, Megumu Yano, Tomomi Imamura, Michiko Akita, Norikazu Tanino, Keisuke Fukuo

**Affiliations:** 1 Department of Food Sciences and Nutrition, School of Food Sciences and Nutrition, Mukogawa Women’s University, 6-46 Ikebiraki-cho, Nishinomiya, Hyogo 663-8558 Japan; 2 Research Institute for Nutrition Sciences, Mukogawa Women’s University, Nishinomiya, Hyogo Japan; 3 Department of Dietary Life and Food Sciences, Junior College Division, Mukogawa Women’s University, Nishinomiya, Hyogo Japan; 4 Department of Innovative Food Sciences, School of Food Sciences and Nutrition, Mukogawa Women’s University, Nishinomiya, Hyogo Japan

**Keywords:** Aging, Physical activity, SARS-Cov-2 outbreak, Sitting time, Social capital

## Abstract

**Background:**

The coronavirus disease 2019 (COVID-19) has adversely affected social contact and physical activity. This study investigated the correlation between physical activity, social contact, and sedentary time among adults aged 65 years and above during the COVID-19 pandemic.

**Methods:**

This study was conducted in N City, H Prefecture, Japan. The authors randomly selected 4,996 adults, aged 65 years and above (mean age 74.1 ± 6.1 years), living in N City, and survey forms were distributed by mail in mid-August 2020. Altogether, 1,925 participants were included in this study. The survey comprised questions concerning the participants’ sex, height, weight, age, smoking and drinking habits, living arrangements, social contact assessments, physical activity levels, and sedentary time. Moreover, linear regression analysis was utilized to investigate the associations between the variables.

**Results:**

The reported median physical activity was 1272 metabolic equivalent of task-min/week (interquartile range 528–2628), and the reported median sedentary time was 360 min/week (interquartile range 240–600). COVID-19 “somewhat,” “quite a lot,” or “completely” hindered the frequency of in-person contact with friends among 75.5% of the respondents and hampered the frequency of virtual contact with friends among 38.8% of the respondents. Physical activity was associated significantly with in-person contact indicators: “interaction with friends” (B = -0.111; 95%CI: -0.187, -0.035; p = 0.004) and “social participation” (B = -0.163; 95%CI: -0.248, -0.079; *p* < 0.001). These associations remained significant for both multivariate analysis Models 1 (sex and age) and 2 (addition of body mass index [BMI], alcohol use, smoking, living alone, and the number of illnesses to Model 1). Additionally, sedentary time was significantly associated with the social contact variable of “interaction with friends” (B = 0.04; 95%CI: 0.016, 0.064; p = 0.001). This association remained significant in both multivariate analysis models.

**Conclusions:**

Significant associations were confirmed between reduced social contact, decreased physical activity, and more sedentary behavior among older adults due to COVID-19. Hence, continuous monitoring and support for social activities among susceptible older adults in extraordinary circumstances are essential.

## Background

The novel coronavirus disease 2019 (COVID-19) spread rapidly across the globe, significantly affecting daily lives [1]. Lockdowns have been imposed in major cities worldwide. A state of emergency was declared on April 16, 2020, in Japan, and activities were restricted for approximately one month. After May 25, 2020, the restrictions were relaxed every few weeks, during which people were only permitted to leave their residences to go to work, buy essential items, or exercise. People were instructed to avoid crowded places, large gatherings, and meetings. Additionally, social activities for older adults were postponed to prevent the spread of COVID-19 amongst the vulnerable segments of society. A recent survey administered by the Survey of Health, Ageing and Retirement in Europe (SHARE) revealed that older adults living with chronic conditions were significantly more likely to be hospitalized because of COVID-19 [2]. As of December 2020, Japan reported 164,203 COVID-19 cases and 2,419 deaths, with an overall mortality rate of 1.3%. While the mortality rate is 0% for those in their 20 s and 30 s, it reached 0.1%, 0.3%, 1.5%, 5.3%, and 12.9% for those in their 40 s, 50 s, 60 s, 70 s, and 80 s, respectively [3]. Thus, the importance of such confinement measures was warranted, especially among older adults.

In Japan, the restrictions due to COVID-19 lasted from April to May 2020; however, social activities for older adults were postponed to prevent the spread of infection [4]. Due to the low-level information communication technology (ICT) utilization of older adults [5, 6], there were concerns that many older adults were at an increased risk of loneliness or social isolation during this time.

Social isolation due to the COVID-19 pandemic has been a public health concern for older adults living in communities since the outbreak of the disease. Social isolation is defined as a state of little to no interaction with family and friends and non-participation in social activities [7]. Social isolation is a high-risk factor for all-cause mortality [8–11] and coronary vascular disease [12–17]. Moreover, social isolation has been associated with depression [18], dementia, and declining cognitive functioning [19–21].

Although the mechanism whereby social isolation influences physical and mental health is not clearly understood, the leading theory is that social isolation results in poor health habits, such as physical inactivity [22, 23]. A recent study of participants aged 50 years and older found that social isolation was associated with low daily physical activity and sedentary behavior [24].

Furthermore, close friendships were associated with more physical activity and less sedentary behavior among older adults [25]. A study on adults aged 62 years and above signified that interaction with friends was positively associated with objective physical activity [26]. Hence, it appears that social contact is critical in increasing motivation to engage in physical activity [27].

Physical inactivity, due to COVID-19 lockdowns and restrictions on activities, has been widely reported globally. Although rates vary by generation and region, studies show a 10‒50% decrease in overall physical activity [28–31] and nearly a 30% increase in sedentary behavior [32, 33]. Even though the restrictions in Japan only lasted for one month, the reduction of social contact due to COVID-19 likely continued to influence older adults’ physical activity until August 2020. To curb the spread of infection, people voluntarily avoided social activities and large gatherings.

A lack of social contact due to the COVID-19 pandemic hindered physical activity among older adults. Therefore, older adults were likely living in extraordinary circumstances even three months after the restrictions were lifted. Accordingly, this research intends to clarify the reduction of social contact among older adults living in communities and explore the correlation between their physical activity and social contact during the COVID-19 pandemic.

## Methods

### Study participants and study period

This study was conducted in Japan in August 2020. In total, 4,996 community-dwelling older people were selected randomly as prospective study participants from an overall older population aged 65 years and above using addresses recorded in the Health and Welfare Department Office. The exclusion criteria included older people who were hospitalized or who resided in nursing homes, and the survey forms were distributed by mail in mid-August 2020. The details of the study were explained in writing to the prospective participants, and the return of a completed survey was considered as consent to participate in the study.

**Fig. 1:**
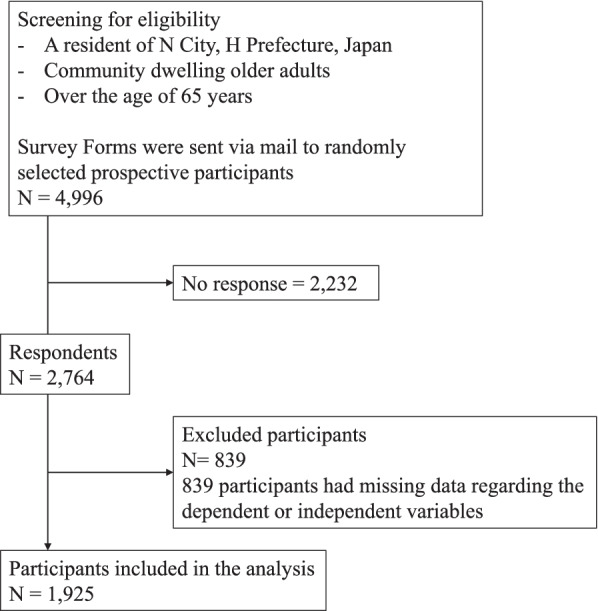
Flowchart for the recruitment of study participants

Survey responses were received from 2,764 participants. Among these, 839 respondents had missing data for sex (*n* = 1), age (*n* = 21), body mass index (BMI) (*n*  = 42), smoking (*n*  = 37), drinking (*n*  = 4), lifestyle (*n*  = 16), International Physical Activity Questionnaire (IPAQ) (*n*  = 16), or social contact (*n* = 559), and were consequently excluded. Thus, a final sample of 1,925 respondents was utilized in the analysis. A flow chart describing the selection process of this study sample is shown in Fig. 1. Additionally, the Ethics Committee of Mukogawa Women’s University approved this study (Approval Number: 20–53).

### Survey content

The survey constituted demographic questions regarding sex, height, weight, age, smoking habits (smoking or non-smoking), drinking habits (drinking alcohol once per week and more, or not drinking at all), and living arrangements (living alone or living with others). Moreover, participants’ self-assessed weights and heights were used to calculate their BMI. Chronic conditions were assessed as self-reported hypertension, diabetes, hyperlipidemia, stroke, cardiac disease, and others.

### Assessment of social contact status due to COVID-19

The modified versions of the question “During the past four weeks, to what extent has your physical health or emotional problems interfered with your normal social activities with family, friends, neighbors, or groups?” from the Short Form 36 Health Survey Questionnaire (SF-36) were employed in this research [34]. In particular, the participants were asked the following questions: “To what extent has COVID-19 interfered with your in-person contact with friends?” “To what extent has COVID-19 interfered with your in-person contact with family?” “To what extent has COVID-19 interfered with your social participation?” Social participation was defined as participation in one or more of the following eight variables: neighborhood associations, senior citizen clubs, hobby groups, sports groups or clubs, political organizations or groups, industrial or trade associations, religious organizations or groups, volunteer groups, and citizen or consumer groups. Participants were also asked the following questions: “To what extent has COVID-19 interfered with your contact through phone, mail, or email with friends?” “To what extent has COVID-19 interfered with your contact through phone, mail, or email with family?” These questions were answered using one of six responses: “Did not interfere at all,” “Interfered very little,” “Interfered somewhat,” “Interfered quite a lot,” “Interfered completely,” or “I do not have friends or family/I do not participate socially.”

### Assessment of physical activity

The IPAQ was utilized to gauge physical activity [35, 36] by asking participants about their duration and frequency of walking, moderate physical activity, and intense physical activity. The IPAQ question “How much time did you spend sitting on a weekday?” was employed to evaluate participants’ self-assessed sedentary time.

### Statistical analysis

IBM SPSS 25.0 was used to perform the statistical analyses. Categorical data are shown as the number and percentages of respondents, whereas continuous variables are shown as mean and standard deviation. Linear regression analysis was utilized to explore the associations between social contact, physical activity, and sedentary time. Both physical activity and sedentary time underwent logarithmic transformation before statistical analysis. Social contact was based on six categories: “Did not interfere at all,” “Interfered very little,” “Interfered somewhat,” “Interfered quite a lot,” “Interfered completely,” or “I do not have friends or family/I do not participate socially.” For associations between social contact and physical activity using linear regression analysis, Model 1 was adjusted for sex and age, whereas Model 2 added adjustments for BMI, alcohol use, smoking, living alone, and the number of illnesses. For associations between social isolation and sedentary time using linear regression analysis, Model A was adjusted for sex and age, whereas Model B added adjustments for BMI, alcohol use, smoking, living alone, and the number of illnesses. Lastly, a two-tailed *p* < 0.05 was considered statistically significant.

## Results

The participants’ demographic data and characteristics are exhibited in Table 1. Notably, 44.2% and 55.8% of the participants were men and women, respectively. The mean age was 74.1 ± 6.1 years, while the median physical activity and median sedentary time were 1272 MET-minutes/week (interquartile range 528–2628) and 360 min/week (interquartile range 240–600), respectively.

**Table 1:** Participants’ characteristics and demographics ^a^

Gender			
Male		851 (44.2)	
Female		1074 (55.8)	
Age,mean ± SD	years	74.1 ± 6.1	
< 75		1120 (58.2)	
≥ 75		805 (41.8)	
BMI	kg/m^2^	22.5 ± 3.1	
< 18.5		156 (8.1)	
≥ 25		353(18.3)	
Lifestyle			
Living alone		359 (18.6)	
Living with others		1566 (81.4)	
Illnesses			
< 2		1110 (57.7)	
≥ 2		815 (42.3)	
Alcohol use			
Does not drink		978 (50.8)	
Drinks		947 (49.2)	
Smoking			
Does not smoke		1769 (91.9)	
Smokes		156 (8.1)	
Physical activity,mdian[IQR]	MET-min / week	1272 [528 1272]	
Sedentary time,mdian[IQR]	min	360 [240 360]	

^a^Values are n(%))

Table 2 presents the status of social contact due to COVID-19, which interfered “somewhat,” “quite a lot,” or “completely” with the frequency of in-person contact with friends in 75.5% of the participants and with the frequency of contact through phone, mail, or email with friends in 38.8% of the participants. Meanwhile, COVID-19 interfered “somewhat,” “quite a lot,” or “completely” with the frequency of in-person contact with family in 65.1% of the respondents and the frequency of contact by phone, mail, or email with family in 30.7% of the respondents. Finally, COVID-19 interfered with social participation “somewhat,” “quite a lot,” or “completely” in 91.8% of the participants.

**Table 2:** Status of participants’social contact due to COVID-19

		Not at all	Verry little	Somewhat	Quite a lot	Completely	Do not have friends or family/Do not participate socially
In-person contact	Friends	149(10.5)	198(14.0)	320(22.6)	456(32.2)	293(20.7)	509
	Family	307(18.7)	264(16.1)	430(26.2)	407(24.8)	231(14.1)	286
	Social participation	33(3.4)	47(4.8)	167(17.0)	331(33.8)	402(41.0)	945
Contact by phone	Friends	669(44.3)	266(16.9)	336(21.3)	230(14.6)	46(2.9)	348
	Family	938(52.7)	297(16.7)	336(18.9)	149(8.4)	60(3.4)	145

Note: Values are n(%)

Table 3 depicts the associations between social isolation and physical activity in a multivariate linear regression analysis. Among the social contact indicators, “in-person contact with friends” (B = -0.111; 95%CI: -0.187, -0.035) and “social participation” (B = -0.163; 95%CI: -0.248, -0.079; p < 0.001) were significantly associated with lower physical activity and remained significant for multivariate analyses of both Models 1 and 2.

**Table 3:** Linear regressions of associations social contact and physical activity

		B	95.0% CI		P
			Lower	Upper	value
Crude	In-person contact with friends	-0.111	-0.187	-0.035	0.004
	Contact by phone, mail, e-mail with friends	-0.047	-0.113	0.018	0.158
	In-person contact with family	0.024	-0.046	0.093	0.505
	Contact by phone, mail, e-mail with family	-0.029	-0.104	0.045	0.445
	Social participation	-0.163	-0.248	-0.079	P < 0.001
	(Constant)	-0.041	-0.106	0.025	0.227
Model 1	In-person contact with friends	-0.118	-0.194	-0.043	0.002
	Contact by phone, mail, e-mail with friends	-0.041	-0.106	0.025	0.227
	In-person contact with family	0.017	-0.053	0.086	0.638
	Contact by phone, mail, e-mail with family	-0.024	-0.099	0.05	0.522
	Social participation	-0.171	-0.255	-0.087	P < 0.001
	(Constant)	8.245	7.647	8.843	P < 0.001
Model 2	In-person contact with friends	-0.133	-0.209	-0.058	0.001
	Contact by phone, mail, e-mail with friends	-0.027	-0.093	0.038	0.416
	In-person contact with family	0.009	-0.06	0.078	0.799
	Contact by phone, mail, e-mail with family	-0.015	-0.089	0.059	0.689
	Social participation	-0.147	-0.231	-0.063	0.001
	(Constant)	8.227	7.562	8.893	P < 0.001

Model 1: adjusted for sex and age

Model 2: added adjustment for BMI, alcohol use, smoking, living alone, and number of illnesses to Model 1

Table 4 highlights the associations between the status of social contact and sedentary time. Among the social contact indicators, “in-person contact with friends” (B = 0.04; 95%CI: 0.016, 0.064; p < 0.001) was significantly associated with higher sedentary time and remained significant for multivariate analyses of both Models A and B.

**Table 4:** Linear regressions of associations between social contact and sedentary time

		B	95.0% CI		P
			Lower	Upper	value
Crude	In-person contact with friends	0.040	0.016	0.064	0.001
	Contact by phone, mail, e-mail with friends	-0.003	-0.024	0.018	0.803
	In-person contact with family	0.001	-0.021	0.023	0.934
	Contact by phone, mail, e-mail with family	-0.006	-0.03	0.018	0.638
	Social participation	0.011	-0.016	0.038	0.424
	(Constant)	5.669	5.514	5.825	P < 0.001
Model A	In-person contact with friends	0.042	0.018	0.067	0.001
	Contact by phone, mail, e-mail with friends	-0.008	-0.029	0.014	0.478
	In-person contact with family	0.003	-0.019	0.026	0.766
	Contact by phone, mail, e-mail with family	-0.008	-0.032	0.016	0.531
	Social participation	0.012	-0.015	0.039	0.387
	(Constant)	5.764	5.572	5.957	P < 0.001
Model B	In-person contact with friends	0.045	0.021	0.07	P < 0.001
	Contact by phone, mail, e-mail with friends	-0.008	-0.029	0.013	0.465
	In-person contact with family	0.004	-0.018	0.026	0.717
	Contact by phone, mail, e-mail with family	-0.008	-0.032	0.016	0.502
	Social participation	0.010	-0.018	0.037	0.489
	(Constant)	5.834	5.618	6.05	P < 0.001

Model A: adjusted for sex and age

Model B: added adjustment for BMI, alcohol use, smoking, living alone, and number of illnesses to Model A

## Discussion

This study investigated the relationship between the reduction of social contact due to the COVID-19 pandemic and physical inactivity among older adults, revealing that most community-dwelling older adults’ participation in social activities and in-person contact with friends and family was limited, even three months after the restriction period had ended. Furthermore, among the indicators of social contact, the reduction of participation in social activities and in-person contact with friends due to COVID-19 are negatively associated with physical activity. Conversely, only in-person contact with friends was positively associated with the participant’s physical activity level and reduced sedentary time. These findings indicate that most of the older adults in our study experienced a reduction in their social contact due to COVID-19, and individuals who reported the greatest reductions also had the lowest physical activity levels and the highest sedentary behavior levels. Therefore, informal social contact can be a crucial health behavior for older adults. These results are consistent with previous studies that reported a protective association between individual-level social capital and physical activity [25]. Although a large-scale study showed an increase in physical activity among older adults from 1987 to 2017 [37], pandemic-related restrictions have likely put an end to this trend.

In-person contact is essential for increasing motivation for physical activity, as confirmed by a meta-analysis study showing that face-to-face or group gatherings were more motivational for physical activity interventions than other deliveries, such as telephone conversations or mail [27]. During the COVID-19 pandemic, people were told to avoid large gatherings and meetings that were not part of their own families. As social activities for older adults were also postponed to prevent the spread of the disease, older adults were not as motivated to engage in healthy behaviors (e.g., physical activities).

The cessation of social activities due to the COVID-19 pandemic is detrimental to the health of older adults in the future. In Japan, the cessation of social activities due to a large-scale natural disaster had drastic effects on older adults’ physical functions. A Japanese study by Tomata et al. reported that older adults residing in areas affected by the Great East Japan Earthquake had a higher disability prevalence three years after the disaster compared with older adults in other unaffected regions [38].

Restricting social activities for older adults to prevent the spread of infection and limiting in-person contact with friends and other people outside their homes facilitates physical inactivity among older adults. An increase in physical inactivity is known to accelerate the risk of diseases, such as frailty and declining cognitive function. Moreover, studies have shown that the effects of a natural disaster on physical function among older adults persist for up to three to six years [38–42]. Thus, it is essential to continue monitoring the physical effects of social isolation among older adults.

### Limitations

Although this study used a large-scale sample of 1,925 older adults, it has several limitations. First, the physical activity survey did not assess the physical activity status before the restriction period; hence, the study cannot clarify the changes in physical activity before and after the restriction periods. Second, only 1,925 of the 2,794 respondents, all from the same region and city, were included in the analysis. Thus, it is difficult to generalize the results of the study. Third, subjective responses were employed to investigate existing illnesses. Fourth, psychological assessments (e.g., for depression) were not performed. Psychological illnesses have been reported to have a significant effect on physical activity; therefore, confounding variables may have interfered with the outcomes. Additionally, unmeasured confounding bias is still plausible because the data were only stratified for basic variables. Reverse causation is also possible because of the cross-sectional design of this study. Fifth, the assessment of physical activity was subjective and there is a need for an objective assessment in the future. Sixth, social desirability bias may have influenced responses concerning family, friends, social participation, or physical activity. Finally, no timeframe was indicated for the question: “To what extent has COVID-19 interfered with your normal in-person contact with friends?” The participants may have responded differently if they considered various time frames (i.e., during and after, or simply during the restriction period), which could have resulted in the overestimation or underestimation of the association between social isolation status and physical activity.

## Conclusions

The greater the reduction in social contact due to the pandemic, the more likely older adults were to be less physically active and sedentary. Therefore, conducting a mid-to-long-term survey of social contact levels among older people and examining the correlation between social contact levels and physical activity levels is imperative. Additionally, physical activity campaigns and support are warranted in vulnerable populations to maintain good health during such crises.

## Data Availability

The data that support the findings of this study are not publicly available because they contain information that could compromise the privacy of the research participants. The data are available from the corresponding author, N.Otaki, upon reasonable request.

## Abbreviations

COVID-19: Coronavirus disease 2019
BMI: Body Mass Index
SHARE: The Survey of Health, Ageing, and Retirement in Europe
ICT: Information Communication Technology
IPAQ: International Physical Activity Questionnaire
SF-36: Short Form 36 Health Survey Questionnaire

## References

[1] World Health Organization. WHO coronavirus disease (COVID-19) dashboard. https://covid19.who.int/. Accessed 26 Jun 2020.

[2] López-Bueno R, Torres-Castro R, Koyanagi A, Smith L, Soysal P, Calatayud J (2022). Associations between recently diagnosed conditions and hospitalization due to COVID-19 in patients aged 50 years and older- A SHARE-based analysis. J Gerontol A Biol Sci Med Sci.

[3] MHLW. Novel coronavirus (COVID-19)|Ministry of health, labor and welfare. https://www.mhlw.go.jp/stf/seisakunitsuite/bunya/0000164708_00079.html. Accessed 28 Jan 2021.

[4] Kobe Shimbun. What are the health implications due to the COVID-19 pandemic? https://www.kobe-np.co.jp/news/hanshin/202008/0013607714.shtml (Article) (in Japanese) Accessed 16 Aug 2020.

[5] Cotten SR, Ford G, Ford S, Hale TM (2014). Internet use and depression among retired older adults in the United States: a longitudinal analysis. J Gerontol B Psychol Sci Soc Sci.

[6] Cabinet Office. Annual report on the Ageing Society; 2018. https://www8.cao.go.jp/kourei/english/annualreport/2018/2018pdf_e.html. Accessed 2 Mar 2021.

[7] de Jong GJ, Havens B (2004). Cross-national comparisons of social isolation and loneliness: introduction and overview. Can J Aging.

[8] Elovainio M, Hakulinen C, Pulkki-Råback L, Virtanen M, Josefsson K, Jokela M (2017). Contribution of risk factors to excess mortality in isolated and lonely individuals: an analysis of data from the UK Biobank cohort study. Lancet Public Health.

[9] Holt-Lunstad J, Smith TB, Baker M, Harris T, Stephenson D (2015). Loneliness and social isolation as risk factors for mortality: a meta-analytic review. Perspect Psychol Sci.

[10] Steptoe A, Shankar A, Demakakos P, Wardle J (2013). Social isolation, loneliness, and all-cause mortality in older men and women. Proc Natl Acad Sci U S A.

[11] Eng PM, Rimm EB, Fitzmaurice G, Kawachi I (2002). Social ties and change in social ties in relation to subsequent total and cause-specific mortality and coronary heart disease incidence in men. Am J Epidemiol.

[12] Holt-Lunstad J, Smith TB (2016). Loneliness and social isolation as risk factors for CVD: implications for evidence-based patient care and scientific inquiry. Heart.

[13] Valtorta NK, Kanaan M, Gilbody S, Ronzi S, Hanratty B (2016). Loneliness and social isolation as risk factors for coronary heart disease and stroke: systematic review and meta-analysis of longitudinal observational studies. Heart.

[14] Udell JA, Steg PG, Scirica BM, Smith SC, Ohman EM, Eagle KA (2012). Living alone and cardiovascular risk in outpatients at risk of or with atherothrombosis. Arch Intern Med.

[15] Heffner KL, Waring ME, Roberts MB, Eaton CB, Gramling R (2011). Social isolation, C-reactive protein, and coronary heart disease mortality among community-dwelling adults. Soc Sci Med.

[16] Barth J, Schneider S, Von Känel R (2010). Lack of social support in the etiology and the prognosis of coronary heart disease: a systematic review and meta-analysis. Psychosom Med.

[17] Kaplan GA, Salonen JT, Cohen RD, Brand RJ, Syme SL, Puska P (1988). Social connections and mortality from all causes and from cardiovascular disease: prospective evidence from eastern Finland. Am J Epidemiol.

[18] Cruwys T, Dingle GA, Haslam C, Haslam SA, Jetten J, Morton TA (2013). Social group memberships protect against future depression, alleviate depression symptoms and prevent depression relapse. Soc Sci Med.

[19] Evans IEM, Martyr A, Collins R, Brayne C, Clare L (2019). Social isolation and cognitive function in later life: a systematic review and meta-analysis. J Alzheimers Dis.

[20] Cacioppo JT, Hughes ME, Waite LJ, Hawkley LC, Thisted RA (2006). Loneliness as a specific risk factor for depressive symptoms: cross-sectional and longitudinal analyses. Psychol Aging.

[21] Cacioppo JT, Hawkley LC (2009). Perceived social isolation and cognition. Trends Cogn Sci.

[22] Berkman LF, Glass T, Brissette I, Seeman TE (2000). From social integration to health: Durkheim in the new millennium. Soc Sci Med.

[23] Hemingway H, Marmot M (1999). Evidence based cardiology: psychosocial factors in the aetiology and prognosis of coronary heart disease. systematic review of prospective cohort studies. BMJ..

[24] Schrempft S, Jackowska M, Hamer M, Steptoe A (2019). Associations between social isolation, loneliness, and objective physical activity in older men and women. BMC Public Health.

[25] Van Holle V, Van Cauwenberg J, De Bourdeaudhuij I, Deforche B, Van de Weghe N, Van Dyck D (2016). Interactions between neighborhood social environment and walkability to explain Belgian older adults’ physical activity and sedentary time. Int J Environ Res Public Health.

[26] Ho EC, Hawkley L, Dale W, Waite L, Huisingh-Scheetz M (2018). Social capital predicts accelerometry-measured physical activity among older adults in the U.S.: a cross-sectional study in the National Social Life, Health, and Aging Project. BMC Public Health.

[27] Knittle K, Nurmi J, Crutzen R, Hankonen N, Beattie M, Dombrowski SU (2018). How can interventions increase motivation for physical activity? A systematic review and meta-analysis. Health Psychol Rev.

[28] Browne RAV, Macêdo GAD, Cabral LLP, Oliveira GTA, Vivas A, Fontes EB (2020). Initial impact of the COVID-19 pandemic on physical activity and sedentary behavior in hypertensive older adults: an accelerometer-based analysis. Exp Gerontol.

[29] Yamada M, Kimura Y, Ishiyama D, Otobe Y, Suzuki M, Koyama S (2020). Effect of the COVID-19 epidemic on physical activity in community-dwelling older adults in Japan: a cross-sectional online survey. J Nutr Health Aging.

[30] Srivastav AK, Sharma N, Samuel AJ (2021). Impact of coronavirus disease-19 (COVID-19) lockdown on physical activity and energy expenditure among physiotherapy professionals and students using web-based open E-survey sent through WhatsApp, Facebook and Instagram messengers. Clin Epidemiol Glob Health.

[31] Akbari HA, Pourabbas M, Yoosefi M, Briki W, Attaran S, Mansoor H (2021). How physical activity behavior affect well-being, anxiety and sleep quality during COVID-19 restrictions in Iran. Eur Rev Med Pharmacol Sci.

[32] Trabelsi K, Ammar A, Masmoudi L, Boukhris O, Chtourou H, Bouaziz B (2021). Globally altered sleep patterns and physical activity levels by confinement in 5056 individuals: ECLB COVID-19 international online survey. Biol Sport.

[33] Trabelsi K, Ammar A, Masmoudi L, Boukhris O, Chtourou H, Bouaziz B (2021). Sleep quality and physical activity as predictors of mental wellbeing variance in older adults during COVID-19 lockdown: ECLB COVID-19 international online survey. Int J Environ Res Public Health.

[34] Fukuhara S, Bito S, Green J, Hsiao A, Kurokawa K (1998). Translation, adaptation, and validation of the SF-36 Health Survey for use in Japan. J Clin Epidemiol.

[35] Craig CL, Marshall AL, Sjöström M, Bauman AE, Booth ML, Ainsworth BE (2003). International physical activity questionnaire: 12-country reliability and validity. Med Sci Sports Exerc.

[36] Murase N, Katsumura T, Ueda C, Inoue S, Shimomitsu T (2002). Validity and reliability of Japanese version of International Physical Activity Questionnaire. J Heal Welf Stat.

[37] López-Bueno R, Smith L, Tully MA, Shin JI, Calatayud J, López-Sánchez GF (2021). Increase in regular leisure-time physical activity in Spanish adults Between 1987 and 2017. Am J Prev Med.

[38] Tomata Y, Suzuki Y, Kawado M, Yamada H, Murakami Y, Mieno MN (2015). Long-term impact of the 2011 Great East Japan Earthquake and tsunami on functional disability among older people: a 3-year longitudinal comparison of disability prevalence among Japanese municipalities. Soc Sci Med.

[39] Tomata Y, Kakizaki M, Suzuki Y, Hashimoto S, Kawado M, Tsuji I (2014). Impact of the 2011 great east Japan earthquake and tsunami on functional disability among older people: a longitudinal comparison of disability prevalence among Japanese municipalities. J Epidemiol Commun Health.

[40] Gero K, Hikichi H, Aida J, Kondo K, Kawachi I (2020). Associations between community social capital and preservation of functional capacity in the aftermath of a major disaster. Am J Epidemiol.

[41] Pruchno R, Wilson-Genderson M, Heid AR, Cartwright FP (2020). Type of disaster exposure affects functional limitations of older people 6 years later. J Gerontol A Biol Sci Med Sci.

[42] Inoue Y, Jeong S (2020). Did the number of older people requiring long-term care and expenditure increase after the 2011 great east Japan earthquake? Analysis of changes over six years. Int J Environ Res Public Health.

